# Emerging Molecular Markers Towards Potential Diagnostic Panels for Lupus

**DOI:** 10.3389/fimmu.2021.808839

**Published:** 2022-01-13

**Authors:** Gongjun Tan, Binila Baby, Yuqiu Zhou, Tianfu Wu

**Affiliations:** ^1^ Department of Clinical Laboratory, Zhuhai Maternal and Child Healthcare Hospital, Zhuhai, China; ^2^ Department of Biomedical Engineering, University of Houston, Houston, TX, United States

**Keywords:** omics, biomarker panel, SLE, disease monitoring, lupus nephritis, neuropsychiatric lupus (NPSLE)

## Abstract

Systemic lupus erythematosus (SLE) is a multifactorial autoimmune disease which can affect various tissues and organs, posing significant challenges for clinical diagnosis and treatment. The etiology of SLE is highly complex with contributions from environmental factors, stochastic factors as well as genetic susceptibility. The current criteria for diagnosing SLE is based primarily on a combination of clinical presentations and traditional lab testing. However, these tests have suboptimal sensitivity and specificity. They are unable to indicate disease cause or guide physicians in decision-making for treatment. Therefore, there is an urgent need to develop a more accurate and robust tool for effective clinical management and drug development in lupus patients. It is fortunate that the emerging Omics have empowered scientists in the discovery and identification of potential novel biomarkers of SLE, especially the markers from blood, urine, cerebrospinal fluids (CSF), and other bodily fluids. However, many of these markers have not been carefully validated for clinical use. In addition, it is apparent that individual biomarkers lack sensitivity or specificity. This review summarizes the sensitivity, specificity and diagnostic value of emerging biomarkers from recent studies, and discusses the potential of these markers in the development of biomarker panel based diagnostics or disease monitoring system in SLE.

## 1 Introduction

A Biomarker is generally defined as a measurable physical, genetic, biological, or biochemical factor that can reflect normal or abnormal biological process when altered. A biomarker should indicate changes that associate with the pathological features and/or presentations of a disease with diagnostic or prognostic potential. They are a crucial component of personalized medicine (1). An optimal biomarker with good sensitivity and specificity can be readily measured in patient-derived samples that are ideally obtained in a minimally invasive way—such as blood, urine or other body fluids. The tests for biomarkers should be reliable, reproducible, and affordable.

Systemic lupus erythematosus (SLE) is a complex autoimmune disease that can affect multiple organ systems and exhibits various signs and symptoms, hence posing significant challenges in diagnosis and treatment. SLE is defined by the detection of elevated autoantibodies in circulation (2) along with abnormal presentation of B and T lymphocytes (3). SLE etiology is multifactorial, contributed by environmental, stochastic, and genetic factors (4). Kaul A et al. stated “Genetic interactions along with environmental factors, particularly UV light exposure, Epstein-Barr virus infection, and hormonal factors might initiate the disease, resulting in immune dysregulation at the level of cytokines, T cells, B cells and macrophages” (5). Consequently, this results in a breach in immune tolerance where the T cells identify self-antigens and deliver assistance to the auto-reactive B cells. These B cells generate a diversified repertoire of autoantibodies. The SLE autoantibodies are able to minimize the extent of organ damage by forming immune complexes by binding to host tissue which are then deposited in vascular tissue resulting in the activation of the immune system. SLE affects various organs, however the most common ones are the kidneys, lungs, skin, joints, components of blood, as well as the central nervous system. Disease severity, treatment response, as well as the array of clinical involvement differs from patient to patient posing considerable challenges in the diagnosis and control of SLE (6).

At present, the criteria for diagnosis of lupus is based primarily on the presence of clinical manifestations in the form of joint pain, skin rashes, glomerular nephritis, symptoms of neuropsychiatric illnesses as well as the results of lab tests such as the presence of antinuclear antibodies, ANA and anti-dsDNA antibodies in particular. The EULAR/ACR classification criteria for SLE (2019) necessitates at least one positive ANA for entry (6), followed by additive weighted criteria which is grouped in seven clinical domains (constitutional, hematological, neuropsychiatric, mucocutaneous, serosal, musculoskeletal, and renal) and three immunological domains (antiphospholipid antibodies, complement proteins, SLE-specific antibodies) that are weighted from 2 to 10 points. Patients that accumulate 10 or more points are classified. In the validation cohort, the new criteria had a sensitivity of 96.1% and specificity of 93.4% (6).

**Figure 1:**
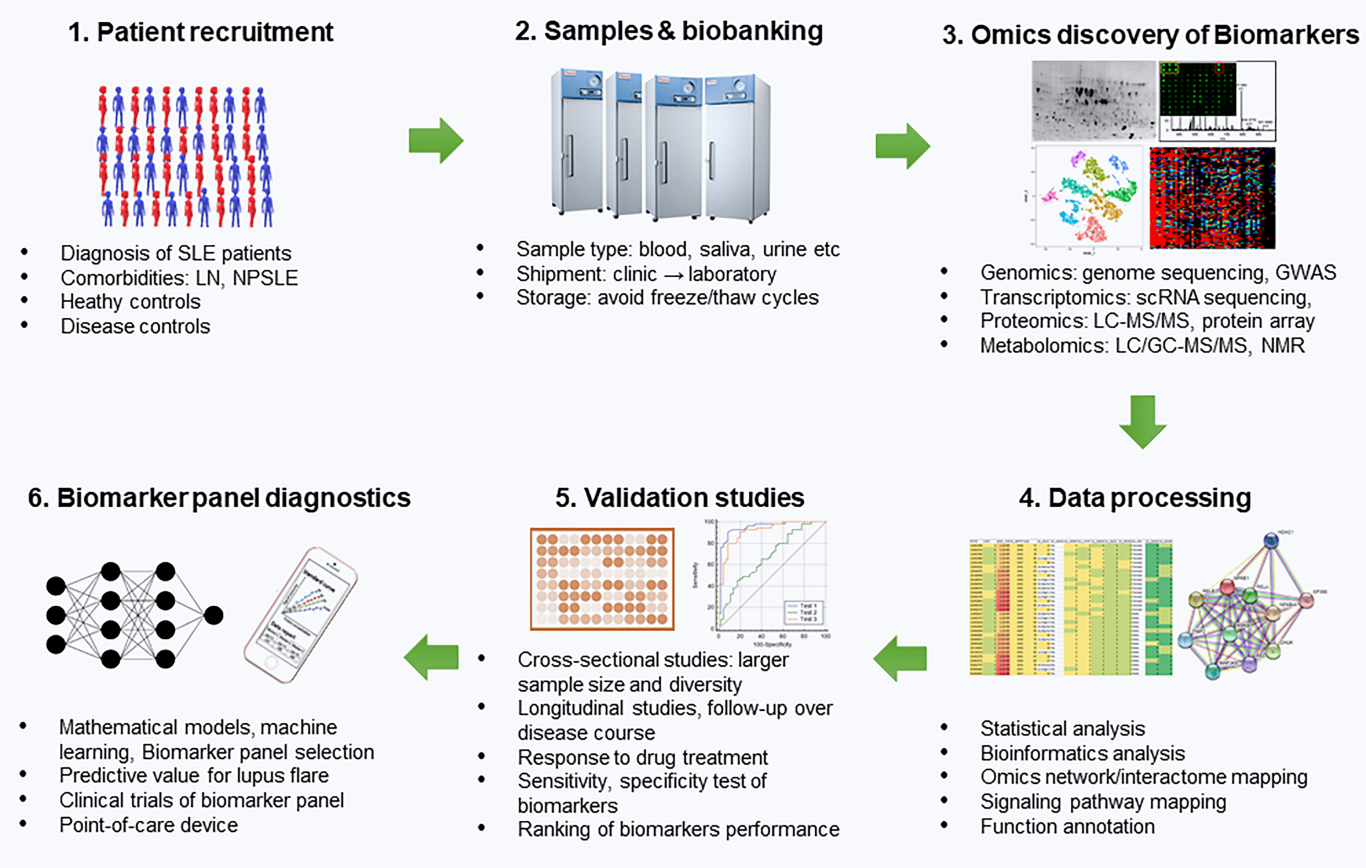
Development of Biomarker panels for predicting lupus flares. This includes patient recruitment, sample collection, biobanking, Omics-based biomarker discovery, statistical and bioinformatics analysis of the potential biomarkers, validation studies using a cross-sectional cohort and a longitudinal cohort, ranking of biomarker performance and selection of biomarker panel using mathematical models and machine learning, clinical trials of biomarker panel and the development of biomarker panel based point-of-care devices for disease monitoring of lupus patients. SLE, systemic lupus erythematosus; LN, lupus nephritis; NPSLE, neuropsychiatric SLE; GWAS, genome-wide-association studies; scRNA, sequencing single cell; RNA, sequencing; LC, liquid chromatography; MS, mass spectrometry; NMR, Nuclear magnetic resonance.

However, currently accessible laboratory markers for SLE diagnosis are suboptimal. Such is the case for the ANA test which has high overall sensitivity (94%) but comparatively low specificity (61%) (7, 8). On the other hand, anti-dsDNA and anti-Sm antibody have good specificity for SLE but low sensitivity as a result of its transient presence (8). To achieve better therapeutic outcomes, it is necessary to continuously assess and monitor the disease progression as well as predict the future disease course. There is a need for more accurate and robust biomarkers for SLE to monitor the disease progression, evaluate treatment response, and predict future flares in an organ-specific manner. As outlined in **Figure 1**, the development of biomarkers or a biomarker panel for predicting lupus flare-ups include patient recruitment, sample collection, biobanking, Omics-based biomarker discovery, statistical and bioinformatics analysis of the potential biomarkers, validations studies using a cross-sectional cohort and a longitudinal cohort, ranking of biomarker performance and selection of biomarker panel using mathematical models and machine learning, clinical trials of biomarker panel, and the development of biomarker panel based point-of-care devices for disease monitoring of lupus patients. This review includes a summary of the recent findings of biomarkers in SLE, and a discussion of their advantages and limitations, especially their potential utility in the future of lupus treatment. We reviewed the biomarkers derived from biological fluids including serum or plasma, CSF, and urine based on sensitivity, specificity and Area-under-the-curve (AUC) as demonstrated by Receiver Operating Characteristic (ROC) curve analysis.

In this review, by using the following keywords: “SLE” or “Systemic lupus erythematosus”, “detection” or “diagnosis”, “biomarker” or “marker” and “AUC” or “ROC” to search the Pubmed, we retrieved “255” relevant research articles. Among these, we filtered out 193 papers which didn’t include an analysis of biomarker performance such as sensitivity, specificity and statistical significance (p-value), or they did not satisfy the following criteria: the candidate biomarkers exhibited an overall AUC > 0.8 with p-value <0.05 and sample size > 10 per group. The final 62 papers were selected to perform further comparison analysis on those promising biomarker candidates as presented in **Tables 1**–**4**.

**Table 1a:** Emerging diagnostic markers of SLE.

Marker	Specimen	Number	Method	Sensitivity	Specificity	AUC	p-Value	Reference
Anti-collectin 11	serum	30/90(SLE/NSLE)	ELISA	n/a/	n/a/	0.806	P<0.001	(9)
Anti -(P0)-4	serum	50/25^#^	Protein array	n/a/	n/a/	0.91	P < 0.05	(10)
Anti (P0)-11	serum	50/25^#^	Protein array	n/a/	n/a/	0.90	P < 0.05	(10)
Anti-DNA topoisomerase 1	serum	50/25^#^	Protein array	n/a/	n/a/	0.93	P < 0.05	(10)
Anti U1-SnRNP 68/70	serum	50/25^#^	Protein array	n/a/	n/a/	0.91	P < 0.05	(10)
Anti HIST1H4A-IgG	serum	153/81^#^	ELISA	95%	90%	0.97	p<0.001	(11)
Anti-alpha-1,6-glucan	serum	30/30^#^	ELISA	93.3%	73.3%	0.863	p = 0.000	(12)
Anti-Tyro3	serum	70/70^#^	ELISA	n/a/	n/a/	0.871	p < 0.0001	(13)
ENA6 sm	serum	30/30^#^	ELISA	70%	100%	0.844	P < 0.001	(14)
Sm/RNP	serum	30/30^#^	ELISA	75%	100%	0.942	P < 0.0005	(14)
Jo-1	serum	30/30^#^	ELISA	83%	90%	0.915	P < 0.0005	(14)
SCL-70	serum	30/30^#^	ELISA	96%	80%	0.899	P < 0.0005	(14)
Angiostatin	urine	100/21^#^	ELISA	n/a/	n/a/	0.93	P<0.0001	(15)
BCDF	serum	36/24^#^	ELISA	80.6%	70.8%	0.861	p < 0.001	(16)
C3dg	plasma	169/170^#^	ELISA	84%	94%	0.96	p < 0.001	(17)
C3dg/C3	plasma	169/170^#^	ELISA	67%	97%	0.89	p < 0.001	(17)
Cyr61	serum	110/100^#^	ELISA	n/a/	n/a/	0.830	P < 0.001	(18)
FAS	serum	28/9^#^	Protein array	n/a/	n/a/	0.91	P < 0.01	(19)
IFI27	blood	61/20^#^	PCR	n/a/	n/a/	91.08	P < 0.01	(20)
IGFBP2	serum	28/9^#^	Protein array	n/a/	n/a/	0.97	P < 0.01	(19)
IgM	serum	36/24^#^	ELISA	97.2%	87.5%	0.902	p < 0.001	(16)
MMP-9	serum	36/30^#^	ELISA	97.2%	n/a/	0.984	P<0.001	(21)
MMP10	serum	28/9^#^	Protein array	n/a/	n/a/	0.91	P < 0.01	(19)
MLKL	blood	59/30^#^	PCR	81.36%	93.3%	0.928	P < 0.05	(22)
OPN	serum	28/9^#^	Protein array	n/a/	n/a/	1.00	P < 0.01	(19)
S100A4	plasma	52/43^#^	ELISA	95.5%	93.0%	0.989	P < 0.001	(23)
S100A12	plasma	52/43^#^	ELISA	70.5%	83.7%	0.807	P < 0.001	(23)
Sema3A	serum	170/150^#^	ELISA	80.6%	77.5%	0.876	P<0.01	(24)
suPAR	plasma	89/29^#^	ELISA	82.02%	79.31%	0.85	p = 0.0001	(25)
Siglec5	serum	28/9^#^	Protein array	n/a/	n/a/	0.96	P < 0.01	(19)
sTNFR1	serum	28/9^#^	Protein array	n/a/	n/a/	0.99	P < 0.01	(19)
sTNFRII	serum	28/9^#^	Protein array	n/a/	n/a/	1.00	P < 0.01	(19)
GAS5	plasma	163/80^#^	qRT-PCR	65.03%	93.75%	0.819	P = 0.003	(26)
circPTPN22	PBMCs	49/37^#^	qRT-PCR	n/a/	n/a/	0.918	P < 0.001	(27)
circRNA407176	PBMCs	122/102 ^#^	qRT-PCR	76.90%	76.90%	0.806	P<0.001	(28)

^#^SLE vs health controls; SLE/NSLE, SLE vs rheumatoid arthritis (RA); primary Sjogren’s Syndrome (SS) and healthy control (HC); n/a, data not available.

**Table 1b:** Emerging activity markers of SLE.

Marker	Specimen	Number	Method	Sensitivity	Specificity	AUC	p-Value	Reference
Ang2	serum	43/30(A/S)	ELISA	81%	89%	0.88	P < 0.001	(29)
CXCL13	serum	36/18(A/S)	ELISA	n/a/	n/a/	0.829	P<0.001	(30)
CXCL13	serum	50/30(A/S)	ELISA	100%	96%	0.989	P < 0.01	(31)
Galectin-9	serum	50/27(A/S)	ELISA	84%	72%	0.84	P < 0.001	(32)
IP-10	serum	27/19(A/S)	ELISA	81.5%	73.7%	0.807	p<0.0001	(33)
IL-17	serum	72/70(A/S)	ELISA	93.3%	92.9%	0.95	P<0.001	(34)
IL-6	serum	72/70(A/S)	ELISA	90.%	90.5%	0.93	P<0.001	(34)
PGLYRP2	serum	30/15(A/S)	ELISA	n/a/	n/a/	0.841	P < 0.01	(35)
PTX3	plasma	64/60(A/S)	ELISA	100%	80%	0.92	P < 0.05	(36)
sTim-3	serum	93/22(A/S)	ELISA	75.3%	81.8%	0.85	p<0.0001	(37)
miR-181a	serum	64/36(A/S)	qRT-PCR	n/a/	n/a/	0.885	P < 0.05	(38)
miR-203	serum	64/36(A/S)	qRT-PCR	n/a/	n/a/	0.843	P < 0.05	(38)
circ_0082689	PBMCs	24/114(A/S+H)	qRT-PCR	87.5%	89.1%	0.913	<0.0001	(39)
circ_0082688	PBMCs	24/114(A/S+H)	qRT-PCR	91.6%	80%	0.924	<0.0001	(39)

A/S, active SLE vs stable SLE; A/S+H, active SLE vs (stable SLE, HC); n/a, data not available.

**Table 2:** Molecular markers of lupus nephritis.

Marker	Specimen	Method	Number	Sensitivity	Specificity	AUC	p-Value	Study
Anti-dsDNA	Serum	IDIM	16/25	56.25%	88%	0.705	p =0.0294	(40)
Anti-dsDNA	Serum	ELISA	227/53	65%	65%	0.75	p< 0.001	(41)
Anti-nucleosome	Serum	IDIM	16/25	87.5%	75%	0.807	p =0.0012	(40)
Anti-C1q	Serum	IDIM	16/25	68.75%	84%	0.843	p =0.003	(40)
AαA	Serum	ELISA	40/40	60%	90%	0.701	P=0.001	(42)
Anti-α-enolase	Serum	ELISA	144/70	82.2%	90.5%	0.809	P=0.004	(43)
AGP	Urine	ELISA	98/30	n/a	n/a	0.87	P<0.02	(44)
Ang-2	Serum	ELISA	60/21	58.1%	90.5%	0.748	p=0.002	(45)
Angiostatin	Urine	ELISA	227/53	82%	80%	0.87	P< 0.001	(41)
Angiostatin	Urine	ELISA	42/12	n/a	n/a	0.97	P<0.001	(46)
APRIL	Urine	ELISA	46/15	n/a	n/a	0·781	P < 0·05	(47)
APRIL	Serum	ELISA	47/27	65%	87.5%	0.713	P<0.05	(48)
BAFF	Urine	ELISA	46/15	n/a	n/a	0·825	P < 0·05	(47)
C24:1Cer	Plasma	LC-MS/MS	46/36	n/a	n/a	0.86	P=0.0001	(49)
C24:1Cer	Serum	LC-MS/MS	46/36	n/a	n/a	0.92	P=0.0001	(49)
C3	Urine	ELISA	227/53	73%	74%	0.82	p< 0.001	(41)
C4d	Serum	ELISA	98/77	79%	58%	0.68	P=0.003	(50)
Ceruloplasmin	Urine	ELISA	98/30	n/a	n/a	0.73	P<0.05	(44)
Ceruloplasmin	Urine	ELISA	76/44	n/a	n/a	0.86	p<0.001	(51)
CXCL4	Urine	ELISA	227/53	61%	63%	0.64	P=0.003	(41)
DKK-1	Serum	ELISA	111/70	77.4%	42.5%	0.783	p = 0.045	(52)
Eotaxin	Serum	Milliplex map	80/40	n/a	n/a	0.777	P < 0.001	(53)
HE4	Serum	ELISA	44/30	81.8%	53.3%	0.714	P < 0.05	(54)
HE4	Serum	ELISA	209/32	76.8%	91.1%	0.878	P < 0.001	(55)
IGFBP-2	Serum	ELISA	87/20	n/a	n/a	0.97	P < 0.0001	(56)
IL-17	Serum	ELISA	80/20	n/a	n/a	0.91	P < 0.001	(57)
IL-17	Urine	ELISA	50/20	66.7%	72%	0.717	P=0.006	(58)
IL-23	Serum	ELISA	80/20	n/a	n/a	0.78	P < 0.01	(57)
IP-10	Serum	ELISA	78/58	n/a	n/a	0.77	p= 0.03	(59)
L-PGDS	Urine	ELISA	98/30	n/a	n/a	0.79	P<0.009	(44)
MCP-1	Urine	ELISA	121/20	n/a	n/a	0.75	p < 0.01	(60)
MCP-1	Serum	ELISA	121/20	n/a	n/a	0.43	P<0.001	(60)
MCP-1	Urine	ELISA	47/53	90%	79%	0.87	<0.001	(61)
MCP-1	Urine	ELISA	78/58	93.3%	53.1%	0.78	p= 0.03	(59)
MCP-1	Urine	ELISA	50/20	76.9%	80%	0.869	P=0.000	(58)
NGAL	Urine	ELISA	54/36	98%	100%	0.997	P < 0.001	(62)
NGAL	Urine	ELISA	34/12	70.8%	87.5%	0.755	P = 0.013	(63)
NGAL	Urine	ELISA	54/36	98%	100%	0.997	p < 0.001	(62)
NGAL	Urine	ELISA	50/20	79.5%	80%	0.875	P=0.000	(58)
OPG	Urine	ELISA	58/63	n/a	n/a	0.72	p < 0.001	(64)
OX40	Blood	FC	40/20	90%	70%C	0.90	P<0.01	(65)
OX40L	Serum	ELISA	40/20	80%	60%	0.71	P<.05	(65)
PGRN	Urine	ELISA	154/71	100%	100%	1.000	P < 0.001	(55)
PGRN	Serum	ELISA	154/71	60.5%	100%	0.877	P < 0.001	(55)
Plasmin	Urine	ELISA	113/41	100%	69.9%	0.86	p< 0.001	(66)
sICAM-1	Urine	ELISA	92/20	94.5%	78.9%	0.874	P<0.001	(67)
TGF-1	Urine	ELISA	50/20	64%	68%	0.665	P=0.038	(58)
TRAF6	Serum	qPCR	128/30	n/a	n/a	0.897	P<0.001	(68)
Transferrin	Urine	ELISA	98/30	n/a	n/a	0.84	P<0.05	(44)
Transferrin	Urine	ELISA	76/44	n/a	n/a	0.84	p<0.001	(51)
TWEAK	Urine	ELISA	70/20	62.22%	93.33%	0.815	p < 0.0001	(69)
VCAM1	Urine	ELISA	227/53	66%	69%	0.73	p< 0.001	(41)
VCAM-1	Urine	ELISA	42/12	n/a	n/a	0.98	P<0.001	(46)
VCAM-1	Urine	ELISA	92/20	98.2%	66.7%	0.882	P<0.001	(67)
β2-MG	Urine	Immunoturbidimetry	144/70	81.8%	90.0%	0.845	P=0.001	(43)
miR-125a	plasma	qRT-PCR	26/26	92%	34%	0.67	P=0.048	(70)
miR-142-3p	plasma	qRT-PCR	26/26	80%	55%	0.62	P=0.185	(70)
miR-146	plasma	qRT-PCR	26/26	56%	96%	0.75	P=0.005	(70)
miR-155	plasma	qRT-PCR	26/26	88%	67%	0.82	p< 0.001	(70)
MiR-29c	Urine	RT-PCR	32/20	94%	82%	0.946	P < 0.001	(71)
miR-21	Plasma	qPCR	26/26	n/a	n/a	0.912	P < 0.001	(72)
miR-146a	PBMCs	qRT-PCR	128/30	n/a	n/a	0.821	P<0.001	(68)
miR-200b-5p	plasma	qRT-PCR	101/100	n/a	n/a	0.748	p < 0.001	(73)
miR-141-5p	plasma	qRT-PCR	101/100	n/a	n/a	0.748	p<0.001	(73)
miR-200c-5p	plasma	qRT-PCR	101/100	n/a	n/a	0.723	p<0.001	(73)
circRNA002453	plasma	qRT-PCR	59/27	90%	84.1%	0.906	p < 0.001	(74)

FC, flow cytometry; LC–MS/MS, Liquid Chromatography and Mass spectrometry; “n/a”, data not available.

**Table 3:** Molecular markers of neuropsychiatric SLE (NPSLE) or other SLE comorbidities.

Marker	Specimen	Number	Method	Sensitivity	Specificity	AUC	p-Value	Study
α-Klotho	CSF	34/84	ELISA	82.4%	94.0%	0.94	p= 0.0004	(75)
ANRIL	plasma	65/35	ELISA	54%	73%	0.66	P=0.02	(76)
APOA1-AS	plasma	65/35	ELISA	65%	66%	0.72	P=0.003	(76)
CCL21	plasma	9/9	ELISA	88.9%	75%	0.85	P < 0.01	(77)
IL-6	CSF	32/13	ELISA	87.5%	92.3	0.956	p < 0.0001	(78)
IP-10	plasma	9/9	ELISA	66.7%	100%	0.82	P < 0.01	(77)
NOS3-AS	plasma	65/35	ELISA	80%	66%	0.71	P=0.004	(76)
OPN	CSF	18/25	ELISA	70%	100%	0.88	p < 0.05	(79)
S100B	serum	47/20	ELISA	84%	61.5%	0.742	p = 0.021	(80)
S100B	serum	87/25	LIA	73.9%	79.8%	0.77	p = 0.009	(81)

LIA, luminescence immunoassay.

**Table 4:** Examples of Biomarker panels for lupus.

Marker	Number	Method	Sensitivity	Specificity	AUC	p-Value	Study
13S1212Cit3,13S1210	60/50	Microarray	n/a	n/a	0.83	P<0.001	(82)
Peaks m/z: 8595, 7170, 7661, 7740, 5806	27/27	MALDI-TOF-MS	92.6%	92.6%	n/a	n/a	(83)
65 specific peptides	34/58	CE-MS	n/a	n/a	0.99	P<0.001	(84)
AGP, CP	31/60	ELISA	n/a	n/a	0.88	P<0.001	(85)
AGP, CP, LPGDS	31/60	ELISA	n/a	n/a	0.90	P<0.001	(85)
AGP, CP,LPGDS,TF	31/60	ELISA	n/a	n/a	0.92	P<0.001	(85)
AGP, CP, LPGDS, TF, VCAM-1	31/60	ELISA	n/a	n/a	0.92	P<0.001	(85)
AGP, CP , LPGDS , TF , VCAM-1 , MCP-1	31/60	ELISA	n/a	n/a	0.92	P<0.001	(85)
Anti-heparan, anti-histone H2B, anti-vimentin	69/203	Antigen array	n/a	n/a	0.845	P < 0.0001	(86)
Anti-α-enolase,β2-MG	144/70	ELISA	91.9%	93.3%	0.927	P=0.004	(43)
MCP-1, TWEAK	70/20	ELISA	86.67%	80.00%	0.887	p < 0.0001	(69)
OPN, adiponectin	14/75	ELISA	81%	67%	0.75	P=0.003	(87)
Plasmin, TFPI	113/41	ELISA	83.8%	86.4%	0.86	p < 0.001	(66)
PGRN(S+U)	154/71	ELISA	100%	100%	1.00	p<0.001	(88)
PG 27:2, proline	32/26	UltraLC	87.5%	76.9%	0.846	P<0.001	(89)
uTGF-1、uNGAL	50/20	ELISA	64.1%	88%	n/a	p<0.001	(58)
miR-21, miR-423, miR-150	26/26	qPCR	79%	83%	0.93	P <.001	(72)
miR-125a, miR-142-3p, miR-146, miR-155	26/26	qRT-PCR	88%	78%	0.89	p< 0.001	(70)
miR-200b-5p, miR-141-5p, miR-200c-5p	101/100	qRT-PCR	80%	93%	0.936	p <0.001	(73)
lnc0597, GAS5	163/80	qRT-PCR	83.44%	93.75%	0.942	P<0.001	(26)
lnc0597,0640,5150,7074,GAS5	240/120	qRT-PCR	95%	85%	0.966	P<0.001	(90)

MALDI-TOF-MS, matrix-assisted laser desorption/ionization time-of-flight mass spectrometry; CE, capillary Electrophoresis; LC, liquid chromatography; “n/a”, data not available.

## 2 Biomarkers in SLE

Manifestations of SLE are linked with various autoantibodies that result in immune complex formation, deposition, and other immune system processes. The clinical presentation as well as pathogenesis is complex making SLE challenging to understand and define (91). According to the 2019 European League Against Rheumatism/American College of Rheumatology, the classification criteria for SLE had a sensitivity of 96.1% and a specificity of 93.4% using positive ANA as an entry criteria, weighted criteria in 7 clinical areas (neuropsychiatric, hematologic, constitutional, mucocutaneous, serosal, musculoskeletal and renal), 3 immunologic domains (antiphospholipid antibodies, low complements, anti-Sm, and anti-dsDNA as SLE-specific antibodies), and a classification threshold score of ≥10 (out of a theoretical maximum of 51) (6).

ANA has a long history serving as a classical clinical marker for the detection and screening of autoantibodies in autoimmune diseases including SLE, however, the sensitivity as well as accuracy of the ANA tests in diagnosis is not satisfactory due to false positives and negatives in previous reports (92–95). Therefore, standardization of ANA-based diagnostic tests in autoimmune diseases are highly recommended (92, 96), including the integration of immunofluorescence ANA (IFA) test with solid phase assays (SPA) such as bead-based high-throughput and/or multiplexing assays (92, 96). Previous studies established that complement components and anti-dsDNA antibody levels have diagnostic, prognostic and predictive values for SLE even before the first clinical signs of disease exacerbation (97–99).

### 2.1 Autoantibody Markers in SLE

Autoantibodies are a major clinical manifestation and the first serological marker of SLE. In **Tables 1A, B**, we summarized autoantibody biomarkers included in the SLE Classification Criteria as well as recently discovered novel autoantibody biomarkers in SLE. A systematic review of Medline, Embase, and the Cochrane database identified 13,080 patients from 64 studies with ANA reported by immunofluorescence on HEp-2 cells. A meta-regression of the ANA operating characteristics found a sensitivity of 97.8% for ANA ≥ 1:80, supporting the use of ANA as an entry criterion (100). Interestingly, about 180 antibodies against various autoantigens were identified in SLE patients which may also be able to indicate comorbidities (2). When using healthy controls and other disease controls, the specificity of anti-dsDNA in diagnosing SLE reached as high as 100% and 97%, respectively (101). The specificity of anti-Sm was 100% in the diagnosis of SLE (14). A high titer of anti-Sm antibody is highly SLE-specific despite the fact that low-titer anti-Sm antibodies in ELISA have been reported in other diseases (102).

In addition to the above autoantibodies which have been included in the criteria, there are more emerging autoantibodies that have demonstrated potential as biomarkers of SLE. An IgG autoantibody panel against six extractable nuclear antigens (ENA): SS-A (Ro 52, Ro 60), SS-B, Sm, RNP/Sm, Scl-70 and Jo-1, namely “ENA-6 Profile” is beneficial for the diagnosis of systemic autoimmune rheumatic diseases (14). The results revealed anti-Sm/RNP as an important marker for the diagnosis of SLE (AUC = 0.942) with 75% sensitivity and 100% specificity, anti-Jo-1 (AUC = 0.915) with 83% sensitivity and 90% specificity, anti-Scl-70 (AUC = 0.899) with 96% sensitivity and 80% specificity (14). A peptide array screening revealed 4 autoantibodies that were bound by acidic ribosomal phosphoprotein (P0)-4, acidic ribosomal phosphoprotein (P0)-11, DNA topoisomerase 1 (full length)-1, and U1-SnRNP 68/70 KDa-1, respectively. The AUC for diagnosing SLE based on these peptides were 0.91, 0.90, 0.93, and 0.91, respectively (10). Serum anti-collectin11 levels was significantly higher in the SLE group and the AUC was 0.806 for the diagnosis of SLE. Additional analysis showed that the positivity rate of anti-collectin11 was very high in SLE patients for whom both anti-dsDNA and anti-Sm antibody were negative. The nervous system and gastrointestinal system involvement are most common in the patients with positive anti-collectin11 (9). A study revealed that serum anti-ribosomal P protein antibody (anti-P) was positive in 38 out of 102 SLE patients (37.3%), and the specificity of anti-P was 96.1% (103). Another study revealed that the specificity and sensitivity of anti- P for SLE diagnosis were 99.4% and 14.2%, respectively in Caucasians, who were generally associated with lower anti-Rib-P antibody levels (104). IgG autoantibodies to histones H4 (HIST1H4A), H2A type 2-A (HIST2H2AA3) and H2A type 2-C (HIST2H2AC) were analyzed in 153 SLE patients and 81 healthy controls and the results showed that HIST1H4A-IgG was shown to be the marker with the best individual diagnostic performance for SLE vs healthy control (AUC = 0.97, sensitivity of 95% at 90% specificity) (11). Another study demonstrated that SLE patients displayed a higher reactivity with the modified equivalent of histone peptides. Reactivity with H4pac showed both a high sensitivity (89%) and specificity (91%) for SLE, while H2Bpac exhibited a high specificity (96%) but lower sensitivity (69%). Reactivity with H3pme appeared to not be specific for SLE. Anti-H4pac and anti-H2Bpac reactivity demonstrated a strong correlation with disease activity (105). Anti-DNase I antibodies were positive in 35 SLE and 8 control patients, without significant difference between the mean antibody concentrations of the 2 groups. Sensitivity of this test was 64.81%, and specificity 84.62% (106). Anti-alpha-1,6-glucan-IgG levels were significantly elevated in patients with SLE and the sensitivity for detecting SLE was 93.3%, whereas the specificity was 73.3% and the area under the ROC curve was 0.863 (12). Antibodies to cell membrane associated DNA (mDNA) were identified by an indirect immunofluorescence assay using a B cell line fixed but not permeabilized with sensitivity of 65% and specificity of 98% (107). Serum anti-lipocalin IgG levels in patients that have SLE were significantly increased in comparison to patients with RA, pSS, SSc, or healthy controls, efficiently distinguishing SLE from other conditions with 49.5% sensitivity and 90.7% specificity (108).

It is apparent that classical autoantibodies are advantageous for initial testing of lupus in clinical settings; however, individual autoantibodies may not be able to achieve satisfactory sensitivity and specificity at the same time. Therefore, an autoantibody panel or autoantibody array technology may aid in improving lupus diagnostics in the future.

### 2.2 Serum Protein Markers in SLE

Besides autoantibodies, some serum proteins such as cytokines, chemokines, mediators, adhesion molecules, and complement fragments have also been implicated in SLE as potential markers.

#### 2.2.1 Cytokines

Cytokines are known to play a vital part in the pathophysiology and immunology of SLE. Thus, a number of promising cytokines have been investigated as an SLE diagnostic or prognostic biomarker. Pacheco Y et al. reported 8 cytokines: IL-8, G-CSF, IL-12/23p40, IFNα, TNFα, IL-17A, IL-6, and IL-10 that were elevated in SLE compared to healthy controls (HCs) (109). In another study, IL-17 and IL-6 were found to be in significantly higher levels in SLE patients compared to normal subjects and were associated with active lupus nephritis, anemia, and positively correlated with SLEDAI-2k scores. ROC curve analysis for IL-6 and IL-17 indicated the optimal cutoff level was 12.3 pg/ml and 19.7 pg/ml, respectively, with AUC of 0.93 for IL-6 and AUC of 0.95 for IL-17 (34). Pentraxin 3 (PTX3) is a protein that is known to employ anti-inflammatory as well as protective effects in peripheral inflammatory conditions such as infections, acute myocardial infarctions, and inflammation of the lungs (110). The plasma PTX3 concentration was significantly higher in SLE patients than healthy controls and the cut-off value was 2.8 ng/mL in discriminating SLE from healthy controls with high sensitivity (100%) and high specificity (80%) (36). In another study, PTX3 had a cut-off point of 1.96 ng/mL and displayed a sensitivity of 34% and a specificity of 96% (111). Soluble urokinase plasminogen activator receptor (suPAR) is a biomarker of systemic inflammation. The ROC analysis of suPAR resulted in an AUC of 0.85 and a cut-off value of 3.54 ng/mL with a sensitivity of 82.02% and specificity of 79.31% in discriminating SLE patients from healthy individuals (25). B cell differentiating factor (BCDF) plays a vital role in the differentiation of B cells and increased levels of BCDF was observed in SLE patients in comparison to healthy controls. ROC analysis revealed an AUC of 0.861 for BCDF in discriminating SLE from healthy controls with a sensitivity of 80.6% and a specificity of 70.8% (16). In a study, hepatocyte growth factor (HGF) had significantly increased serum levels in SLE patients compared to healthy controls, but the matrix metalloproteinase-9 (MMP-9) had decreased serum levels in SLE patients. Serum level of HGF was significantly decreased after treatment in SLE patients, but serum level of MMP-9 increased (21). The serum level of Cysteine rich 61 (Cyr61) was higher in SLE patients compared to healthy controls; ROC analysis indicated Cyr61 may have predictive value in the diagnosis of SLE with an AUC of 0.830 (18). Serum growth arrest-specific protein (Gas6) levels in SLE patients were higher than in normal controls, and the sensitivity and specificity were 72.7% and 84%, respectively, with a cut-off value of 25.3 ng/mL when discriminating SLE from normal controls (112).

#### 2.2.2 Chemokines

Chemokines are a family of small (8–10 kDa) chemotactic cytokines that regulate the migration patterns and positions of immune cells (113). Chemokines as well as their attached receptors have a significant role in the pathogenesis of SLE in human and mouse models (114). Some chemokines have been shown to perform very well as biomarkers in the diagnosis and prognosis of SLE. Serum IP-10 could differentiate SLE patients from healthy controls with a sensitivity of 76% and specificity of 70%.

#### 2.2.3 Complement Components

The complement system plays a major role in SLE. Considering that most of the complement system is present within plasma and available, it could be suitable as a biomarker for diagnosis or monitoring of disease activity (115). Hypocomplementemia was included in the classification criteria of SLE, but the measurement of C3 or C4 often reflects disease activity poorly as the sensitivity and specificity of C3 for SLE are 80% and 14%, respectively (17). C3dg, an activation fragment of C3 which is generated following complement activation, was found in higher levels in SLE patient’s plasma than in the controls. The ROC analysis indicated that C3dg had an AUC of 0.96, which was superior to C3 in differentiating patients from controls. This suggests that C3dg could be considered as a complement activation measurement for SLE classification criteria (17). Significantly elevated levels of C4d and C3d were detected specifically on T and B-lymphocytes of SLE patients. T-C4d had a sensitivity of 56% and specificity of 80%, and B-C4d had a sensitivity of 60% and specificity of 82% in differentiating SLE from other diseases (116). Complement C4d levels on erythrocytes (EC4d) and B cells (BC4d) were several times higher in SLE patients in comparison to patients with other rheumatic diseases as well as healthy subjects (117).

#### 2.2.4 Other Disease Markers in SLE

Galectin-9 was found to be elevated in patients with SLE, and it correlated with disease activity and tissue factor expression. It correlated well with the IFN score with an AUC of 0.86 (32). The proinflammatory calcium-binding S100 family of proteins plays a pivotal role in the pathogenesis of rheumatic diseases (118). The levels of plasma S100 proteins effectively discriminated between SLE patients and healthy controls, with an AUC of 0.989, 0.678 and 0.807 for plasma levels of S100A4, S100A8/9 and S100A12, respectively, indicating that S100A4 may be a potential diagnostic biomarker for SLE (23). Serum S100B’s protein level was increased in NPSLE, reflecting continuing neurological damage (81).

In a recent protein array based study, 48 proteins were upregulated in the serum of SLE patients. Among these, serum levels of AXL, ferritin, and sTNFRII were significantly elevated in patients with active lupus nephritis (LN) in comparison to dormant SLE patients. Interestingly, OPN, sTNFRI, sTNFRII, IGFBP2, SIGLEC5, FAS, and MMP10 displayed capacity to discriminate SLE from healthy controls with an ROC AUC exceeding 90% (p < 0.001) (19).

Despite the fact that there is not a satisfactory protein biomarker that can be used in clinic for SLE patients, emerging proteomics may bear great promises in screening for potential candidate biomarkers that could eventually be used to develop a biomarker panel with improved sensitivity and specificity in the diagnosis or disease monitoring of lupus. Besides the efforts towards the discovery of novel biomarkers, tremendous work in validation studies will be urgently needed to test if the aforementioned markers could truly reflect disease status, especially in multi-cohorts or multicenter settings with an increased sample size and over a disease course.

### 2.3 MicroRNAs and Long Non-Coding RNAs (LncRNA) as Disease Markers in SLE

Recent studies discovered some microRNAs (miRNAs), negative regulators of protein expression at the post-transcriptional level through mRNA stability reduction and translation inhibition, were closely connected with SLE pathogenesis. Therefore, miRNAs have great potential as diagnostic markers or therapeutic targets of lupus. Circulating miRNAs can easily be identified through non-invasive methods and numerous have been identified as biomarkers of lupus, as summarized in **Tables 1A, B**. In whole peripheral blood, miR-146a and miR-155 were elevated in SLE patients compared to healthy controls (HCs) (119). In a separate study, compared to healthy controls, miR-21, miR-181a and miR-196a were found to be upregulated in SLE patients, with an AUC of 0.73, 0.72 and 0.76, respectively. It was found that miR-196a was a better marker in differentiating SLE patients from healthy controls, whereas miR-21 was a better marker in discriminating mild SLE from severe SLE in patients (120). In another study, plasma miR-21 levels in SLE patients were higher than that of healthy controls, with an AUC of 0.64 when differentiating SLE from healthy controls (121).

Besides microRNA markers, others found dysregulated expression of circRNAs or lncRNAs involved in the pathogenesis of autoimmune diseases (**Table 1**). The levels of hsa_circRNA_407176 and hsa_circRNA_001308 were decreased in both plasma and peripheral blood mononuclear cells **(**PBMCs) in SLE when compared with healthy controls. In plasma, the AUC of hsa_circRNA_407176 and hsa_circRNA_001308 were 0.599 and 0.662, respectively (28). However, in PBMCs, the AUC of hsa_circRNA_407176, hsa_circRNA_406567, and hsa_circRNA_001308 were 0.806, 0.744, and 0.722, respectively. The study demonstrated that hsa_circRNA_407176 and hsa_circRNA_001308 in plasma and PBMCs could be potential biomarkers for SLE (28). Plasma levels of GAS5 and lnc-DC were significantly decreased in SLE patients compared to healthy controls, while linc0597 was overexpressed in SLE patients; the combination of GAS5 and linc0597 provided better diagnostic accuracy with an AUC of 0.942 (26). Plasma levels of linc0597, lnc0640, and lnc5150 were found elevated, but GAS5 and lnc7074 levels were decreased in SLE patients compared to HCs. The combination of five lncRNAs achieved an AUC ranging from 0.604 to 0.833 when compared to healthy controls in an independent validation phase. This panel of five lncRNAs had high diagnostic accuracy for SLE (AUC = 0.966) and distinguished SLE from RA and pSS (AUC = 0.683 and 0.910, respectively) (90). Miao et al. found patients with higher SLEDAI scores had lower expression levels of circPTPN22, and long-term hormone treatment had significantly increased circPTPN22 levels. ROC curve analysis indicated that circPTPN22 had good diagnostic value for SLE (27). These findings suggest that circulating miRNA, lncRNA and other RNA or DNA fragments in the blood stream may hold great promise as biomarkers for lupus. They are relatively easy to detect using standard polymerase chain reaction (PCR), which is cheaper compared to protein biomarkers assays. However, the unstable nature of these molecules may compromise the accuracy of detection in some cases.

### 2.4 Disease Activity Markers in SLE

The diagnostic markers of SLE are mainly discussed above, and the markers in this section are disease activity makers, which are mainly used to distinguish active SLE or flare from inactive SLE or to determine disease activity of SLE. Based on the definition by Lupus Foundation of America, “Flare is a measurable increase in disease activity in one or more organ systems involving new or worse clinical signs and symptoms and/or laboratory measurements. It must be considered clinically significant by the assessor and usually there would be at least consideration of a change or an increase in treatment” (122). Circulating angiopoietin2 (Ang2) levels were increased in patients with active SLE compared to healthy controls. A calculated Ang2 cut-off value of >2.0 ng/ml was obtained with a specificity of 89% and sensitivity of 81% in discriminating active from inactive SLE (29). A proliferation-inducing ligand (APRIL) in the serum as well as its intrarenal mRNA levels were associated with resistance to treatment. The serum levels of APRIL at 4 ng/ml could accurately predict the response to treatment with a sensitivity of 65% and a specificity of 87.5% (48). High circulating Osteopontin (OPN) levels preceded increased cumulative disease activity and organ damage in SLE patients, especially in pSLE (123). Serum protein, CXC ligand 13 (CXCL13), plays a key role in chemotaxis of B cells; its levels in SLE patients were significantly increased. The ROC analysis demonstrated that serum CXCL13 level could be useful in identifying active disease from overall SLE patients with considerable accuracy (AUC = 0.829) (30). At a cutoff level of 80 pg/mL, CXCL13 could discriminate active SLE from inactive (AUC = 0.989, sensitivity = 100%, specificity = 96%) (31). Serum and urinary IP-10 levels were found to be significantly elevated in active SLE patients compared to inactive SLE patients with a sensitivity of 81% and specificity of 71% (124). At the optimal cutoff point of 14.41 pg/ml of IP-10, the AUC for IP-10 serum levels that differentiated active pediatric systemic lupus erythematosus (pSLE) from inactive pSLE was 0.807 with a sensitivity of 0.815 and specificity of 0.737 (33). Serum leucine-rich α2-glycoprotein (LRG) was found to be higher in patients with active SLE compared to inactive SLE and healthy controls. Serum LRG significantly correlated with SLEDAI-2K and clinical laboratory variables. ROC analysis revealed that optimal serum LRG cutoff value for active SLE was >45.7 ng/ml, and the AUC of LRG for predicting active SLE was 0.666 (125). Hyperprolactinemia is prevalent in SLE patients and correlated with clinical disease activity and the urine protein-creatinine index (UPCI). An ROC curve analysis of serum prolactin could predict SLE disease activity with a sensitivity of 91.7%, specificity of 58.1%, and AUC of 0.74 (4). In another study, SLE patients exhibited significantly higher serum levels of miR-181a and lower serum levels of miR-203, which were correlated with SLE disease activity (126). The results suggested both miR-181a and miR-203 have diagnostic values for active SLE, with an AUC of 0.885 and 0.843, respectively (126). These disease activity biomarkers, if validated, may have great potential in monitoring disease activity or predicting lupus flare.

## 3 Disease Markers in Lupus Nephritis

Lupus nephritis (LN), one of the most common and serious clinical manifestations of SLE, is a leading cause of mortality and morbidity. Various novel immunosuppressive drugs and biological therapies have improved SLE/LN survival rates, however early diagnosis and consistent monitoring of disease flares are still urgently needed for a better therapeutic outcome. The gold standard for diagnosis and prognosis of LN in modern medicine is renal biopsy; however, it should not be used for routine or repeated monitoring of disease activity and treatment response due to its invasive nature (66). During these past years, emerging studies have focused on screening and searching for non-invasive biomarkers which could reflect renal pathology or disease activity in LN, as summarized in **Table 2**.

### 3.1 Autoantibodies in LN

Classical autoantibodies, including anti-dsDNA, anti-cardiolipin, anti-ribosomal P, anti-SSA/Ro, anti-Sm, anti-endothelial cells, anti-epithelial cells, anti-glomerular matrix, and anti-glomerular basement membrane (GBM) antibodies have been found to be associated with LN (3). Pesickova et al. found that anti-CRP antibodies were detected solely in patients that had active renal disease and levels of antibody present correlated with SLEDAI (127). Anti-C1q antibody was found to have a strong association with LN (40). When anti-C1q was greater than 134 U/ml, there was a 15-fold increased risk of LN, with a specificity of 92% and sensitivity of 56%. Serum alpha-actinin antibody (AαA) was significantly lower in LN in comparison to SLE patients without nephritis. Serum AαA at cut-off levels ≤ 59.5 pg/ml could be used to discriminate between the two groups with sensitivity of 60%, specificity of 90%, and positive predictive value of 85.7% (42).

### 3.2 Potential Protein Markers in LN

Besides autoantibodies, some serum protein markers have been indicated to be involved in LN (**Table 2**). Serum human epididymis protein 4 (HE4) levels were significantly higher in LN patients that were positive for anti-dsDNA antibody with low C3. HE4 had a predictive value for LN with an optimal cutoff of 64.8 pM, AUC of 0.714, sensitivity of 81.8%, and specificity of 53.3% according to the ROC curve (54). When the cutoff value was 150.1 pM, the sensitivity and specificity reached 76.8% and 91.1%, respectively in the diagnosis of LN (55). Urinary clusterin was significantly elevated in LN patients with tubulointerstitial renal lesions. ROC curve analysis was used to diagnose the cases who progressed to ESRD, and they found that at the optimal cutoff point of urinary clusterin, the AUC was 0.804 with sensitivity of 72% and specificity of 82% (128). Neutrophil gelatinase-associated lipocalin (NGAL) was identified as an early marker in the kidney after ischemic or nephrotoxic injury. NGAL was easily detected in the urine and blood soon after acute kidney injury, and uNGAL could discriminate patients with nephritis from those without nephritis, with the best cut-off value of 13.66 ng/ml, AUC of 0.959 with sensitivity of 92% and specificity of 75% (129). At the cutoff value of 80 ng/mL, uNGAL levels serve as a predictor for the presence of LN with a high AUC of 0.997 with good sensitivity (98%) and specificity (100%) (62). In an independent study, at a cutoff value of 91.25 ng/mg creatinine, uNGAL had a sensitivity of 0.89 and a specificity of 0.67 (130). C4d levels were significantly increased in patients with SLE. According to ROC curve analysis, C4d levels could discriminate between high and low disease activity exhibiting a positive predictive value of 68% (50). At high disease activity, C4d levels were correlated predominantly with lupus nephritis and exhibited a sensitivity of 79% (50). Complement factor H-related proteins (CFHRs), consisting of proteins CFHR1 through CFHR5, are a part of the broader factor H/CFHR family. The levels of CFHR3 and CFHR5 found in plasma were higher in patients with lupus nephritis than in healthy individuals, and patients with both high CFHR3 and high CFHR5 exhibited the shortest progression-free survival (131).

The levels of IL-17 and IL-23 were found to be higher in patients with active LN compared to patients with inactive LN or healthy controls (57). The AUC of IL-17 to predict the activity of LN (SLEDAI > 9) was 0.91, whereas the AUC of IL-23 to predict the activity of LN (SLEDAI > 9) was 0.78 (57). In LN patients, plasma eotaxin, TNF-α, interleukin-17-α, interleukin-10, and interleukin-15 were significantly increased compared to the SLE non-nephritis group (53). Urine angiostatin displayed higher specificity and sensitivity in discriminating active renal SLE from active non-renal SLE with an AUC of 0.87 and correlated significantly with proteinuria (41). Higher levels of serum and urine Dickkopf-1 (DKK-1) proteins were detected in SLE patients compared to healthy subjects. DKK-1 levels especially were higher in patients with LN in comparison to non-nephritis SLE patients (52). Urine APRIL (uAPRIL) and BAFF (uBAFF) levels were significantly increased in LN, and ROC curve examination of uBAFF and uAPRIL showed an AUC of 0.825 and 0.781, respectively, in distinguishing between nephritic and non-nephritic SLE patients (47). Urinary monocyte chemoattractant protein 1(uMCP-1) level was significantly higher in LN and correlated well with LN disease activity. The cut-off value of uMCP-1 was 82 pg/ml, where AUC was 0.727 with a sensitivity of 88.5% and specificity of 46.3% in identifying LN (132). These levels fell with treatment and could have potential to predict a poor response and subsequent relapse of LN (60). Serum and urine progranulin (PGRN) levels were significantly higher in LN and closely associated with the disease activity of LN (55). Urine CD163 levels were significantly higher in patients with active LN than healthy controls and ROC curves showed an AUC of 0.998 in the predefined groups of active and inactive LN (133).

Urinary osteoprotegerin (uOPG) was significantly higher in active LN. It showed modest correlation with disease activity with a potential to predict poor response to treatment and relapse of LN (64). Urinary and serum IP-10 could be potentially useful markers of lupus activity in differentiating active from inactive lupus, and their AUC was 0.68 and 0.77, respectively (59). Urine plasmin could discriminate active LN from inactive disease with an AUC of 0.84 (66). Some ceramides (Cer) such as C16cer, C18Cer, C20Cer, and C24:1Cer were elevated in serum and plasma samples of patients with LN with impaired renal function compared to healthy controls, as well as non-nephritic SLE patients (49). In this study, C24:1dhCer was implicated as a potent biomarker for renal impairment in patients suffering from SLE (49). Urinary levels of transferrin (TF) and ceruloplasmin (CP) were significantly higher in patients with LN compared to those without LN, with an AUC of 0.84 and 0.86, respectively in discriminating LN from non-LN controls (51). Urinary angiostatin and vascular cell adhesion molecule-1 (VCAM-1) exhibited outstanding potential with an AUC of 0.97 and 0.98, respectively to predict renal biopsy activity index score ≥ 7, which can be associated with poor long-term prognosis (46). However, the urinary angiostatin was not able to discriminate LN patients from other CKD patients with an AUC 0.56 (15). Urinary soluble cellular adhesion molecules (sVCAM-1) and VCAM-1 levels were significantly elevated in LN patients compared to the controls, and the ROC curve of urine sICAM-1 showed an AUC of 0.874 with high sensitivity (0.945) and specificity (0.789), whereas the AUC of VCAM-1 was 0.882 with a sensitivity of 0.982 and specificity of 0.667 (67). The level of urinary transforming growth factor beta 1 (uTGF-β1) and urinary interleukin 17 (uIL-17) were significantly higher in severe LN than control groups. The AUC values of uTGF-β1 and uIL-17 were 0.665 and 0.717, with a cut-off value of 27.13 pg/ml and 36.62 pg/ml, respectively (58). Angiopoietin-2 (Ang2) level was increased in SLE patients in comparison to the control, and it was significantly higher in the LN patients than in SLE patients that did not have nephritis. Ang2 positively correlated with SLEDAI, 24 hours proteinuria, as well as histological activity index (45). Ang2 could indicate the degree of endothelial activation and may potentially be used as a biomarker for both disease activity and renal involvement in SLE patients. However, Ang2 level could not distinguish between proliferative and non-proliferative lesions in LN (45). TNF receptor associated factor 6 (TRAF6) was upregulated in LN patients and was related to LN activity. It positively correlated with serum IL-1β, IL-6, IL-8, as well as TNF-α activity. The AUC of TRAF6 for the diagnosis of LN was 0.897 (68). Stanley et al. found that urinary IL- 7, IL- 12p40, IL- 15, IP- 10 and TARC levels were significantly higher in patients with active LN in comparison to those with inactive SLE as well as healthy controls. It also correlated with renal SLEDAI and physicians global assessment of disease activity (134).

### 3.3 MicroRNA as Disease Markers in LN

A growing body of evidence indicates that microRNAs participate in LN development and kidney fibrosis (135, 136). Since miRNAs are present in body fluids with high stability and can be sampled non-invasively, some of them have been reported as potentially advantageous as diagnostic and prognostic biomarkers for a variety of human diseases (137). Recent findings of miRNAs as potential LN biomarkers were summarized in **Table 2**. For example, miR-146a expression was significantly reduced in LN and was found to be associated with LN activity. The AUC of miR-146a for the diagnosis of LN was 0.821, and the AUC of miR-146a for differentiating LN activity was 0.921 (68). The levels of circulating miR-21 was significantly increased in LN patients compared to healthy controls, and ROC analysis indicated that miR-21 was better at discriminating LN patients from controls with an AUC of 0.912 (72). The multivariate ROC curve analysis showed that the plasma circulating miR-125a, miR-142-3p, miR-146, and miR-155 together could distinguish most of the patients with LN from controls with an AUC of 0.89, sensitivity of 88%, and specificity of 78% (70). Levels of MiR-29c in urinary exosomes displayed a negative correlation with the histological chronicity index as well as glomerular sclerosis. MiR-29c expression levels could predict the degree of chronicity in LN patients with a remarkable AUC of 0.946, sensitivity of 94% and specificity of 82%, respectively (71). Kidney biopsies from LN patients revealed elevated lncRNA RP11-2B6.2 levels and was positively correlated with IFN scores and disease activity (138). There was a statistically significantly decrease in Lnc3643 levels of SLE patients with proteinuria compared with those without (139). LN may be discriminated from SLE without nephritis through lnc-DC (26).

## 4 Disease Markers for Neuropsychiatric SLE (NPSLE) and SLE With Other Comorbidities

### 4.1 Markers for Neuropsychiatric Systemic Lupus Erythematosus (NPSLE)

NPSLE, one of the most serious complications associated lupus, affects both the central and peripheral nervous systems. NPSLE manifestations are associated with varying degrees of morbidity that differ in presentation and severity between patients. They are often times difficult to differentiate from other neuropsychiatric conditions with a different etiology (140). There is no gold standard diagnostic approach for NPSLE that exists. There are however various clinical, laboratory, and radiographic findings reported for differential diagnosis of neuropsychiatric conditions that are associated with SLE. The NPSLE diagnosis remains a challenge — no diagnostic test is available, and differential diagnosis is often obtained through a process of elimination. Many factors contribute to the lack of consistency such as variation in study design, study methodology, patient selection criteria and the rarity of some neuropsychiatric syndromes (140). Increasing interest has been focused on the identifying biomarkers vital to the origin of the disease and as a result, correlated closely with disease activity and outcome. Some potential biomarkers that have been reported in NPSLE patients are summarized below in **Table 3**. The capability for S100B protein levels to differentiate between patients with and without NPSLE was analyzed and it showed good discriminatory capacity for NPSLE (AUC = 0.77) and a better capacity for acute NPSLE (AUC = 0.82). At the cut-off point of 0.125ng/ml, S100B levels would provide a sensitivity of 73.9% and a specificity of 79.8% in differentiating NPSLE. In the case of acute NPSLE, this cut-off value would deliver a sensitivity of 77.8% and a specificity of 79.8% (81). An independent study revealed S100B had a discriminating value for NPSLE patients with peripheral polyneuropathy with an AUC of 0.706 (80). The CSF α-Klotho levels showed decent discriminatory capability for NPSLE (AUC = 0.94). The cut-off point ≤ 230.2 pg/ml would deliver a sensitivity of 82.4% and a specificity of 94.0% for NPSLE (75). By using a Surface-enhanced laser desorption/ionization (SELDI) technique, a panel of m/z peaks at 8595, 7170, 7661, 7740, and 5806 were identified to build a diagnostic decision tree model which could recognize NPSLE with a sensitivity of 92.6% based on training group samples (83). ROC curve analysis showed that the sensitivity and specificity of CSF IL-6 for the diagnosis of lupus psychosis (LP) were 87.5% and 92.3%, respectively, and the AUC was 0.9567 with a cut-off value of 4.3 pg/ml (78). The CSF concentration of OPN was significantly higher in NPSLE than in non-NPSLE. When the cut-off value of OPN in CSF was at 963.4 ng/ml, the sensitivity and specificity for the diagnosis of NPSLE were 70% and 100%, respectively (79). Lipocalin-2 (LCN2) was upregulated in the CSF of NPSLE patients across two different ethnicities, demonstrating that CSF LCN2 may be a novel biomarker for NPSLE (141).

Anti-glyceraldehyde 3-phosphate dehydrogenase (GAPDH) autoantibodies were found to be significantly elevated in SLE patients, particularly in patients with NPSLE (142). Anti-DNA/NR2 antibodies in NPSLE were higher than those in healthy controls, indicating that anti-DNA/NR2 antibodies may be a predictive factor in post-steroid neuropsychiatric manifestation (PSNP) -SLE (143). Anti-Suprabasin (SBSN) antibodies were significantly higher in the CSF of the NPSLE group compared to the non-NPSLE group, indicating that anti-SBSN could potentially be a novel marker for the evaluation of suspicious NPSLE (144). The levels of anti-UCHL-1 autoantibodies in the NPSLE group were significantly higher than in the control group, and the positive rate of anti-ubiquitin C-terminal hydrolase L1 (anti-UCHL-1) autoantibodies in the NPSLE group was 23.7% (145). Significantly higher anti-microtubule-associated protein-2 (anti-MAP-2) antibody titers were discovered in the CSF of patients with NPSLE compared to the CSF of non-NPSLE controls. Anti-MAP-2 antibody prevalence was 33.3% in NPSLE (146).

### 4.2 Disease Markers in SLE With Cardiovascular Diseases (CVD)

Cardiovascular diseases, one of the most serious complications associated SLE, have emerged as a leading cause of illness and mortality. There are several novel biomarkers that have been reported in recent studies in addition to anti-phospholipid antibodies, as summarized in **Table 3**. Some lncRNAs were found to be relevant to atherosclerosis such as antisense lncRNA of INK4b/ARF/INK4a locus (ANRIL), antisense lncRNA of NOS3 (NOS3-AS), and antisense transcript of APOA1 (APOA1-AS) which were increased in atherosclerotic SLE patients than non-atherosclerotic SLE patients. Multivariate analysis identified these lncRNAs as independent predictors for atherosclerosis in SLE (76). SLE patients with a CVD history had higher serum levels of both S100A8/A9 and S100A12 compared to patients without CVD or venous thromboembolism (147). Meta-analysis revealed an increased risk of recurrent major adverse cardiac events in patients with high IgG anti-cardiolipin antibodies both at 12 and 24 months (148). Anti-HDL antibodies were associated with higher risk of CVD in SLE patients, and anti-PON1 antibodies were associated with intima-media thickness in SLE (149). Serum E-selectin was increased in SLE patients and particularly associated with atherosclerosis in patients with SLE (150). Serum annexin A5 was found as an independent predictive variable for endothelial dysfunction in SLE patients (151).

### 4.3 Disease Markers of SLE With Lung Complications

Respiratory tract complications are highly frequent in SLE patients, yet there are only a limited number of studies assessing risk factors or biomarkers that might be able to predict pulmonary manifestations in SLE (SLEpulm), as summarized in **Table 3**. Chemokine (C-C motif) ligand 21 (CCL21) and CXCL10 (IP-10) levels were significantly higher in SLEpulm than SLE without pulmonary manifestations. ROC analysis demonstrated that CCL21 could discriminate SLEpulm from non-pulmonary SLE with an AUC of 0.85, sensitivity of 88.90% and specificity of 75%; likewise, CXCL10 had a good discriminatory value for SLEpulm (AUC = 0.82; sensitivity = 66.67%, specificity% = 100%) (77). Plasma Cyr61 concentration in SLE-associated pulmonary arterial hypertension (SLE-PAH) patients was significantly higher than matched SLE-non-PAH patients and healthy controls. Cyr61 level ≥140.7 pg/ml was indicated to be an independent risk factor for developing PAH in SLE patients (152).

### 4.4 Disease Markers of Cutaneous Lupus Erythematosus (CLE)

Cutaneous lupus erythematosus (CLE) is a frequent manifestation in SLE patients and can also exist as a single entity without associated systemic autoimmunity (153). It can persist for many years and impair quality of life, including vocational disability. Recently, some potential biomarkers have been identified to distinguish CLE from other types of SLE as summarized in **Table 3**. For example, CD40 was intensely expressed in all subacute cutaneous lupus erythematosus (SCLE), discoid LE (DLE), and dermatomyositis (DM) lesions (154). The CCR4 ligand TARC/CCL17 was found to be strongly expressed in skin lesions and its levels were elevated in CLE patient’s serum (155). Soluble E-selectin was significantly elevated in DLE patients with wide-spread lesions and correlated significantly with active cutaneous skin lesions (156). Serum IL-17A and IL-17F concentrations were increased in DLE and SLE patients (157). In addition, anti-C1q antibody levels were correlated with cutaneous Caspase 3 expression in SCLE patients (158). The gene expression of Tyrosine kinase 2 (TYK2), interferon regulatory factor 5 (IRF5), and CTLA4 are associated with SLE and conferred risk for DLE and SCLE (159). SCLE patients had significantly higher levels of anti-C1q antibodies and serum C1q circulating immune complexes (C1q-CIC) levels in comparison to healthy controls. Anti-Laminin-1 antibodies were found in the sera of cutaneous lupus erythematosus patients (160).

## 5 Biomarker Panels for SLE: the Destination

SLE is a complex autoimmune condition affecting multiple organ systems and displays a variety of clinical signs and symptoms. Therefore, it is difficult to accurately diagnose or evaluate the prognosis of SLE with a single biomarker. Combinations of different biomarkers have been explored in the diagnostic or prognostic assessment of SLE to improve its sensitivity and specificity as summarized in **Table 4**. A combination of 13S1212Cit3-IgM with 13S1210-IgG (termed “COPSLE” for the combination of peptides for SLE) was more effective for SLE diagnosis, with an AUC of 0.830 and a positive rate of 73.33%. This combination could be utilized for the identification of 80.0% of SLE patients found negative for anti-Smith, anti-dsDNA, and anti-cardiolipin (ACA) antibodies (82). The combined model of fecal phosphatidylglycerol and proline resulted in an AUC of 0.846 with a good diagnostic value (89). A biomarker panel with 65 peptides were applied to the discovery cohort and resulted in an AUC of 0.99 in discriminating SLE from healthy controls (84). The combination of urine plasmin and tissue factor pathway inhibitor (TFPI) discriminated active LN from inactive LN with an AUC of 0.86, exceeding the specificity as well as positive predictive value of traditional individual markers such as anti-dsDNA and complement C3 (66). The combination of adiponectin and OPN predicted chronic LN damage with an AUC of 0.75, sensitivity of 81% and specificity of 67% (87). The combination of uTGF-β1 and uNGAL exhibited a sensitivity of 64.1% and specificity of 88% for LN (58). The combination of miR-21, miR-423, and miR-146 could differentiate LN from controls with an excellent AUC of 0.93, sensitivity of 79%, and specificity of 83% (72). The combination of plasma circulating miR-125a, miR-142-3p, miR-146, and miR-155 together could distinguish most of the patients with LN from controls with an AUC of 0.89, sensitivity of 88%, and specificity of 78% (70). A combined model of uMCP-1 and uTWEAK showed an AUC of 0.887, sensitivity of 86.67% and specificity of 80.00% to discriminate active LN, and an AUC of 0.778, sensitivity of 75% and specificity of 81.82% to discriminate LN with poor outcome (69). The combination of miR-200b-5p, miR-141-5p, and miR-200c-5p disclosed a greater diagnostic value for LN with an AUC of 0.936, sensitivity of 80%, and specificity of 93% (73). Urinary alpha-1-acid glycoprotein (AGP), ceruloplasmin (CP), VCAM-1, MCP-1, and Lipocalin-like prostaglandin D synthase (LPGDS) levels were significantly higher in those patients with active LN than non-LN patients. The model including both AGP and CP resulted in an AUC of 0.88. With the addition of LPGDS to this model, the AUC increased to 0.90, and further increased to 0.92 upon the addition of TF. The addition of VCAM-1 and MCP-1 into this model however did not increase the AUC (85). The combination of IgG autoantibodies against heparan sulphate, histone H2B, and vimentin could differentiate NPSLE from non-NPSLE with an AUC of 0.845 (86). The combination of urinary VCAM-1, CystatinC, and KIM-1 discriminated proliferative LN from membranous LN with an AUC of 0.80 (95%CI: 0.69–0.90) (161). A combination of five urinary proteins, namely L-PGDS, transferrin, ceruloplasmin, MCP-1, and sVCAM-1 was a good predictor of active LN (AUC= 0.898).A combined model of L-PGDS, transferrin, alpha-1-acid glycoprotein (AGP-1), ceruloplasmin, MCP-1 and sVCAM-1 predicted response to rituximab treatment at 12 months (AUC = 0.818) (162). Proinflammatory high-density lipoprotein (HDL) (piHDL), leptin, plasma soluble TNF-like weak inducer of apoptosis (sTWEAK), and homocysteine when combined with clinical variables such as age and diabetes, could create a risk profile as “predictors of risk for elevated flares, damage progression, and increased cardiovascular disease in patients with SLE (PREDICTS)”. The PREDICTS profile could accurately identify patients with SLE at risk for future subclinical atherosclerosis progression (163).

With the rapid development of single-cell RNA sequencing (scRNA seq) and its application in the profiling of genes associated with SLE or LN (164–166), more novel biomarkers or biomarker panels may be emerging. However, these gene expression data must be validated at the protein level before moving to biomarker detection in clinical settings. It is advantageous to combine multiple LN biomarkers to constitute a biomarker panel to improve the sensitivity or specificity for disease diagnosis, especially in discriminating LN from controls, or discriminating active LN from inactive LN. The reason for this is that different biomarkers represent different biological activity behind LN and collectively they reflect various aspects of this multifactorial disease, hence improving the diagnostic value for LN. However, current biomarker panels are largely limited to a combination of biomarkers from the same categories such as microRNA panel, autoantibody panel, cytokine panel, peptide panel, metabolite panel etc. This is clearly a huge restraint in developing a robust biomarker panel for LN. Moving forward, we should combine the most promising biomarkers across different categories as mentioned above, incorporate pathological markers, and some robust descriptive clinical scores to develop more accurate and clinically useable biomarker panels for LN.

Besides the biomarker panels described in previous studies (**Table 4**), novel biomarker panels may be identified based on future validation results from the promising individual biomarkers as listed in **Tables 1A, B**, for diagnostic a biomarker panel and a disease activity biomarker panel, respectively. For a diagnostic biomarker panel of SLE, based on the preliminary data of the performance of individual markers, anti-HIST1H4A, S100A4, C3dg, TNFRII and IGFBP2 seem to be good candidates if they are validated by other research groups. Likewise, CXCL13, PTX3, IL-6 and IL-17 seem to be promising candidates to constitute a disease activity biomarker panel for SLE if they are validated. In addition, urinary CD163, PGRN, VCAM1, NGAL and Angiostatin seem to have good discriminative capability in the diagnosis and prognosis of active LN. That being said, beyond the biological validity of these biomarker candidates, there are three additional factors impacting the selection of biomarkers for a successful biomarker panel: (1) Technical compatibility of each biomarker within the panel during detection: the ability to detect all biomarkers within the panel in one assay is needed to make the panel viable for clinical use; (2) Availability of high-quality antibodies for each biomarker within the panel: a careful selection of high-quality antibodies with good affinity and specificity is key to build a biomarker panel based assay. (3) A statistically meaningful biomarker panel: it is critical to use multivariable model to generate a statistically sound biomarker panel so that the later stage evaluation of diagnostic or prognostic ability of the panel may have a satisfactory outcome.

## 6 Conclusion

Accurate diagnosis and early treatment can significantly improve therapeutic outcome and prognosis for SLE. Therefore, a good molecular diagnosis is desirable for SLE to reflect disease activity, monitor drug response, and predict flares. Various Omics technologies are promising in identifying novel and robust biomarkers for SLE.

As SLE is a multi-factorial disease with multiple molecular and pathological alterations, individual biomarkers are insufficient in satisfying the clinical need in diagnosis and disease monitoring with desirable sensitivity and specificity. Fortunately, the combination of molecularly and pathologically relevant biomarkers of SLE may significantly improve the accuracy and robustness for disease detection and prediction. Ultimately, the incorporation of these biochemical markers in mainstream clinical care will require validated, standardized laboratory tests that are available worldwide. Such tests need to be robust, reliable, easy-to-perform, and affordable. In addition, standardization of relevant biomarkers must be established, because the relevant biomarker values are usually not very consistent across different studies. For this, the following challenges in LN biomarker studies need to be tackled: (1) The sample size for most studies were relatively small, due to the limited collaborative lupus biomarker research consortium and access to a centralized clinical sample bank. On the other hand, the heterogenous nature of lupus requires a larger sample size in order to identify a statistically meaningful biomarker for SLE and LN. (2) Many of these studies only included healthy donors as controls and fewer studies had disease controls such as other autoimmune diseases or relevant chronic diseases. (3) Commercial ELISA kits have often been directly used as a quantitative method. However, a more careful test, especially the validation of these kits in various sample types by different research groups are still lacking, particularly prior to large-cohort validation studies. Mass spectrometry may be used to further confirm the target biomarker, which is thought to be selectively bound by the capture antibody in the ELISA kit. (4) Autoantibodies and immune complexes are abundant in the blood samples of lupus patients, which may inevitably interfere with the assay *via* competition with capture antibody or detection antibody in the ELISA kit and generate misleading results. All these challenges have to be tackled before a clinically useful biomarker or biomarker panel is identified.

With the development of artificial intelligence and machine learning technology, SLE and LN biomarkers discovered from SLE Omics studies may be categorized according to age, sex, ethnicity, geography, genetic etiology, molecular and cellular mechanism, pathological changes in patients to inform disease cause and drug response, and to guide personalized medication for SLE.

## Author Contributions

GT and TW conceived this work. GT prepared the first draft of the manuscript. BB, YZ, and TW edited the manuscript. All authors contributed to the article and approved the submitted version.

## Conflict of Interest

The authors declare that the research was conducted in the absence of any commercial or financial relationships that could be construed as a potential conflict of interest.

## Publisher’s Note

All claims expressed in this article are solely those of the authors and do not necessarily represent those of their affiliated organizations, or those of the publisher, the editors and the reviewers. Any product that may be evaluated in this article, or claim that may be made by its manufacturer, is not guaranteed or endorsed by the publisher.

## Glossary

**Table:**

AGP	alpha-1-acid glycoprotein
Ang2	Angiopoietin-2
Ang2	angiopoietin2
anti-MAP-2	anti-microtubule-associated protein-2
anti-P	anti-ribosomal P protein antibody
anti-UCHL-1	anti-ubiquitin C-terminal hydrolase L1
APRIL	A proliferation-inducing ligand
AUC	area-under-the-curve
A&alpha;A	alpha-actinin antibody
BAFF	tumor necrosis factor family
BCDF	B cell differentiating factor
CCL21	Chemokine (C-C motif) ligand 21
CFHRs	Complement factor H-related proteins
CLE	Cutaneous lupus erythematosus
CNS	central nervous system
COPSLE	combination of peptides for SLE
CP	ceruloplasmin
CSF	cerebral spinal fluids
CVD	cardiovascular diseases
CXCL13	CXC ligand 13 protein
Cyr61	Cysteine rich 61
DKK-1	Dickkopf-1
GAPDH	glyceraldehyde 3-phosphate dehydrogenase
Gas6	growth arrest-specific protein
HCs	health controls
HE4	human epididymis protein 4
HGF	hepatocyte growth factor
HIST1H4A	histones H4A
IFI27	Interferon Alpha Inducible Protein 27
IP-10 IFN-&gamma	inducible protein 10
IRF5	interferon regulatory factor 5
LCN2	lipocalin-2
LN	Lupus nephritis
LP	lupus psychosis
LPGDS	lipocalin-like prostaglandin D synthase
LRG	leucine-rich &alpha;2-glycoprotein
MCP-1	monocyte chemoattractant protein 1
mDNA	membrane associated DNA
miRNAs	microRNAs
MLKL	Mixed lineage kinase domain-like pseudokinase
MMP-9	matrix metalloproteinase-9
NGAL	Neutrophil gelatinase-associated lipocalin
NPSLE	neuropsychiatric SLE
OPG	Osteoprotegerin
OX40L	ligand for OX40
PG	phosphatidylglycerol
PGRN	progranulin
pSLE	pediatric systemic lupus erythematosus
PTX3	Pentraxin 3
ROC	Receiver Operating Characteristic
SLE	systemic lupus erythematosus
SLE-PAH	systemic lupus erythematosus-associated pulmonary arterial hypertension
suPAR	Soluble urokinase plasminogen activator receptor
TF	transferrin
TFPI	tissue factor pathway inhibitor
TNFSF4/OX40	TNF superfamily member 4
TRAF6	TNF receptor associated factor 6
TYK2	Tyrosine kinase 2
UPCI	urine protein-creatinine index
uTGF-&beta;1	urinary transforming growth factor beta 1
VCAM-1	vascular cell adhesion molecule-1
